# Soil Physicochemical and Biological Properties of Paddy-Upland Rotation: A Review

**DOI:** 10.1155/2014/856352

**Published:** 2014-06-02

**Authors:** Wei Zhou, Teng-Fei Lv, Yong Chen, Anthony P. Westby, Wan-Jun Ren

**Affiliations:** ^1^Key Laboratory of Crop Eco-Physiology and Farming System in Southwest China, Ministry of P.R. China, Wenjiang, Sichuan 611130, China; ^2^College of Agronomy, Sichuan Agricultural University, Wenjiang, Sichuan 611130, China; ^3^Rice Research Institute, Sichuan Agricultural University, Wenjiang, Sichuan 611130, China

## Abstract

Paddy-upland rotation is an unavoidable cropping system for Asia to meet the increasing demand for food. The reduction in grain yields has increased the research interest on the soil properties of rice-based cropping systems. Paddy-upland rotation fields are unique from other wetland or upland soils, because they are associated with frequent cycling between wetting and drying under anaerobic and aerobic conditions; such rotations affect the soil C and N cycles, make the chemical speciation and biological effectiveness of soil nutrient elements varied with seasons, increase the diversity of soil organisms, and make the soil physical properties more difficult to analyze. Consequently, maintaining or improving soil quality at a desirable level has become a complicated issue. Therefore, fully understanding the soil characteristics of paddy-upland rotation is necessary for the sustainable development of the system. In this paper, we offer helpful insight into the effect of rice-upland combinations on the soil chemical, physical, and biological properties, which could provide guidance for reasonable cultivation management measures and contribute to the improvement of soil quality and crop yield.

## 1. Introduction

Green Revolution technologies have allowed the food supply of Asia to satisfy the demand of its rapidly growing population in the past decades; however, the pressure on soil and other resources has intensified [1]. The cultivated area is continuously decreasing because of soil pollution, land abandonment, urbanization, and other reasons [2]. Meanwhile, the population and the demand for food continue to increase. Under such a situation, increasing cropping intensity from monoculture to double or triple cropping in a year is an efficient way to guarantee food security on the amount of agricultural land now available.

Paddy-upland rotation is the most important cropping system in southern and eastern Asian countries such as Bangladesh, China, India, Nepal, and Pakistan [3]. This type of rotation has many different sequences, where numerous grain and industrial crops could be rotated with paddy rice. The rotation between rice and dry season crops has a long history; rice-wheat rotation, which is one of the largest and most important agricultural production systems in the world, started during the Tang Dynasty of China [4]. Rice-wheat rotation, which accounts for approximately 13.5 million ha of the Indo-Gangetic Plains of Bangladesh, India, Nepal, and Pakistan and approximately 10.5 million ha of fields in China, is essential to meeting the food demand of the rapidly increasing human population [5, 6]. However, the current situation is not very optimistic, because the cultivated areas of rice and wheat have declined in the last decade [7]. What is worse, previous study of long-term continuous cropping experiments in Asia has reported the yield stagnation or even declination of rice-wheat cropping system [8].

Many studies on this issue have been conducted because the sustainability of rice-based cropping systems is important to the food security of Asia. Changes in soil properties caused by cultivation and management and their consequences to soil productivity have generated significant research concern for many years. Evidence indicates that the degradation of soil quality is a key factor for the observed declining yield [9]. As a result, researchers have studied the influences of paddy-upland rotation on soil quality and developed effective cultivation and management strategies to sustain soil fertility and maximize crop yield per unit input. In consideration of the unique feature of paddy-upland rotation, all of the soil remediation methods used in this cropping system should consider the different effects on paddy and dry season crops and their interactions with one another. In this study, we reviewed the soil chemical, physical, and biological properties of rice-based cropping systems and identified the aspects that need special attention and consideration to gain guiding references for future research and to contribute to the sustainable development of paddy-upland rotation.

## 2. Soil Physical Properties of Paddy-Upland Rotation

Rice and upland crops are grown annually in sequence influence each other; however, the soil conditions required by rice growth differ from those required by upland crops. Soil is puddled before rice transplanting and kept flooded to create anaerobic conditions for rice growth. By contrast, upland crops are grown in well-drained soil under tillage and aerobic conditions. Several benefits have been found in rice cultivated under puddled condition. Puddling created a plow layer that reduces hydraulic conductivity to support water ponding, which minimized the water percolation losses and enhanced the water and nutrient use efficiency of rice [10]. Puddling triggered a series of changes in soil physical properties. Puddling deteriorated soil physical properties by breaking down soil aggregates, forming hardpans at shallow depth that leaded to induced changes in pore size distribution; the cone index decreased after puddling and gained strength during the subsidence stage of the puddle soil, and the bulk density (BD) of soil increased and hydraulic conductivity decreased 30 and 60 days after puddling [11]. All of the above mentioned changes are believed to have negative effects on the following upland crop [2].

Paddy-upland rotation could change the soil physical properties of long-term-flooded paddy. Studies have indicated that after the application of paddy-upland rotation, the adverse effects in the long-term-flooded paddy fields have shown slight improvements, such as increased soil granular structure and capillary porosity, improved redox potential of soil, and removed secondary gleization. For instance, Huang and Ding [12] showed that soil water-stable aggregate increased by 12.54% after paddy-upland rotation is applied. However, the influence of paddy-upland rotation on BD had different results. Motschenbacher et al. [13] indicated that BD differed among common rice-based cropping systems; however, even after 10 years of continuous production on a silt-loam soil, increased near-surface soil BD has not been achieved. A successive 10-year experiment showed that the BD of the 0 cm to 10 cm layer soil of paddy-upland rotation was 23.4% higher than that of the continuous cropping of semilate rice [14]. BD, which is an indicator of soil quality, increases with time as particles settle after puddling is halted [10]; this indicator is inversely related to many important soil properties, including water-holding capacity, soil particle size, total porosity, infiltration capacity, hydraulic conductivity, gas exchange, and nutrient mobility, which could influence seedling emergence and root penetration. The compaction of paddy soil leads to low germination rates and limits root development in deep soils for subsequent upland crops [15–17]. Data from rice-wheat systems have shown 8% to 9% reduction in wheat yield when wheat was sown after puddled transplanted rice in comparison with being sown after dry direct-seeded rice without puddling. However, the conflicts between puddled rice and succeeding crops are not consistently observed. Farooq et al. [18] concluded that the effect of puddling on succeeding crops varies for different types of soil and crops and pointed out that much of this response inconsistency is likely related to the site-specific nature of soil puddling.

**Figure 1:**
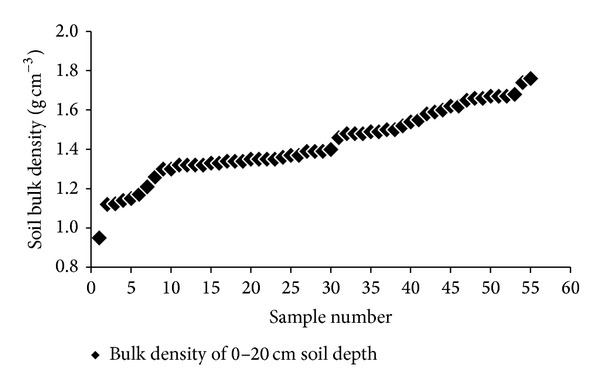
Variation in soil bulk density of paddy-upland rotations. Data are from [2, 13, 14, 19–24].

There is a large variation in soil BD of paddy-upland rotations, which ranges from 0.9 g cm^−3^ to 1.8 g cm^−3^ [2, 13, 14, 19–24] (Figure 1). This difference may be due to different soil types and textures and different cultivation and management measures, such as puddling intensity and depth [25, 26]. Under the condition of paddy-upland rotation, the change of soil physical properties is a complex process, and such change is influenced not only by the soil original properties and crop growth but also by the production and operation practice of farmers. The damaging effect of continuous cultivation with frequent tillage has long been recognized; thus, minimum tillage systems, namely, no-tillage or zero tillage (ZT), are practiced to maintain and improve the soil quality of paddy-upland rotation. Compared with conventional tillage (CT), ZT, which has minimal soil disturbance and soil structure destruction, promotes the formation of macroaggregates, increases water-stable aggregates, aggregate stability, and least-limiting water range, and decreases BD and soil penetration resistance [27, 28]. However, Gathala et al. [2] indicated that ZT in wheat and CT in rice made the benefits of ZT attained during the wheat phase lost in the rice phase. For paddy-upland rotations, ZT technology is more effective for rice production, upland crop production, or both.

## 3. Soil Chemical Properties of Paddy-Upland Rotation

### 3.1. Main Seasonal Changes Driven by Paddy-Upland Rotation

**Figure 2:**
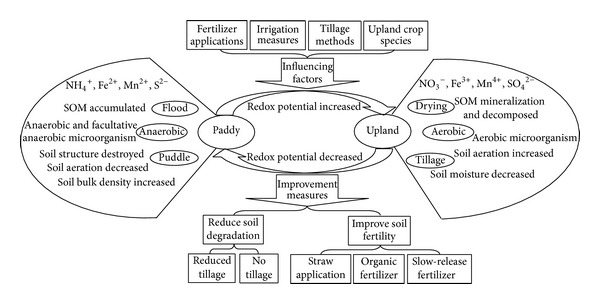
Characteristics of paddy-upland rotation and its improvement measures. SOM: soil organic matter.

Soil fertility must be maintained to sustain and improve long-term agricultural productivity, that is, crop yields. Comparison of soil under natural vegetation and adjoining cultivated topsoil has revealed that prolonged agricultural land use alters the magnitude, diversity, and spatial variability of a number of soil properties, primarily those related to fertility [1]. Paddy-upland rotation fields are unique from other wetland or upland soils, because of the seasonal alternation of wetting and drying and the frequent alternation of anaerobic and aerobic conditions; the chemical speciation and biological effectiveness of soil nutrient elements vary with seasons (Figure 2). Under flooded conditions, the redox potential of paddy is low and NO_3_^−^, Fe^3+^, Mn^4+^, and SO_4_^2−^ are, respectively, reduced to NH_4_^+^, Fe^2+^, Mn^2+^, and S^2−^. Thus, flooding also improves the availabilities of P, K, Si, Mo, Cu, and Co and reduces the availabilities of N, S, and Zn. By contrast, during the upland crop season, the redox potential is increased, thereby oxidizing the soil nutrient elements and changing the effectiveness of the abovementioned elements [4]. Gupta et al. [29] argued that in most lowland rice soils, P availability initially increased on flooding and rice may meet its P requirement from the residual P applied to the receding wheat. Li et al. [30] indicated that the efficiency of K fertilizer application is affected by various factors, and both rice and the subsequent crop remove enormous amounts of K, resulting in a significant negative K balance in soils regardless of whether K fertilizers are applied at recommended doses. Mn deficiency, which is common in the wheat of rice-wheat rotation systems in China [31] and India [32], leads to the decline in wheat yield. Except for the sporadic use of micronutrients of paddy-upland rotation [20], the decrease in Mn availability in upland field is the main reason for the Mn deficiency [33]. The change in soil moisture content also influences soil pH, thereby affecting the chemical equilibrium and consequently changing the form and effectiveness of soil nutrient elements [34].

### 3.2. Soil C and N Cycles under Paddy-Upland Rotation Condition

Soil productivity is closely linked with soil organic matter (SOM) status, which is important for nutrient mineralization, soil structural improvements, and favorable soil-water relations [25]. Soil structural degradation is common in intensively cultivated ecosystems because of SOM depletion [35]. Tiessen et al. [36] reported approximately a 1% loss in organic carbon per year during the first 20 years to 30 years in soils under cereal cultivation. SOM decomposition is generally slower in water-logged soil than in well-aerated soil; however, the frequent cycling between anaerobic and aerobic conditions of paddy-upland rotation results in a greater rate of SOM decomposition [13]. During rice season, the number and activity of reducing bacteria are increasing, which not only leads to the lower decomposition of organic matter and contributes to SOM accumulation but also promotes the production of toxic substances such as CH_4_ [37]. During upland crop season, the biological N fixation is reduced and SOM mineralization is facilitated, thereby accelerating SOM loss [38]. Kumari et al. [39] posited that soil C is incorporated first into macroaggregates (>0.25 mm) and then forms the core of new microaggregates; this physical protection of C within macroaggregates limits its oxidation by creating a less favorable environment for microbial activity and thus reduces its decomposition rate by half or more. As a result, the excessive tillage and extractive farming practices reduce SOC stocks. Imbalanced or inappropriate fertilizer practices and intensive cropping with no return of crop residues and other organic inputs also result in SOM loss [40]. Soil types and the choice of crop species could also affect SOM content. Result shows that lighter soil texture had higher decomposable organic C and total C declined than heavy soils [1].

With its negative effects on the environment, the use of N fertilizers and its cycle has received research interest for several years. N fertilizer application is the most important source of soil N, and the three major paths of N losses (ammonia volatilization, denitrification, and leaching) are all influenced by N application, soil moisture state, and tillage practice; consequently, the paddy-upland rotation significantly affects the soil N cycle because of the contrasting soil and water conditions and different nutrients required by the two crops. Research has confirmed that N_2_O emission mainly occurs during the drying period of soil rather than during the flooding period, indicating that the alternation of wetting and drying could improve N_2_O emission [41]. Other field trials have revealed that the accumulation of inorganic N during upland crop season would be lost to the environment immediately after flooding [42]. Bueno and Ladha [1] found that a clear association exited between organic matter parameters and N uptake and that declining C pools caused lower N uptake. Moreover, soil C and N cycles influence each other through microbiological action.

The interactions between paddy rice and upland crops make the soil C and N cycles more complicated, thereby increasing the difficulty of water and nutrient management of paddy-upland rotation. Paddy-upland rotation is generally harmful for soil C and N storage under the current condition of the cultivation and management practice of farmers. The changes in soil fertilizer before and after experiment under different land utilizing types are shown in Table 1 [14, 19, 20, 40, 43–48]. The effect of the same paddy-upland rotation (rice-wheat) on soil fertilizer varies in different experiments. Compared with other land utilizing types, such as upland-upland rotation, rice-fallow, and fallow, paddy-upland rotation, particularly rice-wheat rotation, is inefficient for maintaining or improving soil fertility. Meanwhile, the rice-Chinese milk vetch rotation could increase soil N content. Researchers have demonstrated that the benefits of legumes in rotation are not only caused by biological N fixation but also by increased nutrient availability, enriched soil fertility, improved soil structure, reduced disease incidence, and increased mycorrhizal colonization, which could help to sustain the long-term productivity of cereal-based cropping systems [49]. Therefore, the rice-wheat cropping system could be diversified by using legumes as substitute crop. The application of organic fertilizers, including green manure, farmyard manure, crop residues or straws, and compost manure, is also an effective measure of improving soil fertility and maintaining land productivity. However, at the present status of organic fertilizer application and the characteristics of nutrient utilization and cycling, three differences should be clarified before using organic fertilizers in paddy-upland rotations: the nutrient content of different organic fertilizers and its release characteristics; the fertilizer requirement rule of different crops, such as require time and amount; and the interactions between rice and different upland crops, such as the influence of residual soil fertility on the following crops. Accordingly, we can guarantee the optimizing timing and doses for using organic fertilizers and ensure the effective utilization of fertilizers.

## 4. Soil Biological Properties of Paddy-Upland Rotation 

Soil microorganisms are involved in various biochemical processes and are vital in maintaining soil fertility and plant yields. The diversity of rhizosphere microorganisms is beneficial to soil health, and the trophic interactions within the rhizosphere affect the aboveground community of plants [50, 51]. Different plants have different soil microbial communities, and crop rotation provides greater concentration and diversity of organic materials, both of which may lead to greater diversity of microbial communities.

Several studies have reported a positive effect of crop rotation on the abundance of beneficial microorganisms. Cropping systems could markedly affect the composition, abundance, diversity, and activity of soil bacterial communities; evidence shows an association between crop type and microbial community composition. For instance,* Bradyrhizobium* sp. and* Herbaspirillum* sp. colonize the interior of rice roots when grown in rotation with a legume crop, which may promote rice growth and productivity [52]. Furthermore, the availability of soil microorganisms increases the availability of plant nutrient elements, especially N and P [53]. Therefore, Xuan et al. [54] indicated that appropriate crop rotation provides a feasible practice for maintaining equilibrium in soil microbial environment for sustainable rice cultivation.

Other effects of crop rotation on soil biological properties are diseases, especially soil-borne diseases, and weed suppression. Different crop species with diverse root exudates and plant residues create varying patterns of resource competition, allelopathic interference, soil disturbance, and mechanical disruptions [55, 56], which lead to an unstable and frequently inhospitable environment where little organisms could survive [57]. Studies have shown that crop rotation is an effective method for reducing crop pests and diseases. The S*clerotinia* stem rot of oilseed rape, which is caused by* Sclerotinia sclerotiorum*, is one of the most common fungal diseases of rapeseed. Ding [58] indicated that sclerotinias could only survive 20 days under flooded condition and that the paddy-upland rotation could reduce the incidence. Yuan et al. [59] showed that the incidence of the disease of sesame-rape rotation was 32.2% higher than that of rice-rape rotation.* Verticillium* wilt, which is caused by soil-borne fungus, is a worldwide disease affecting temperate and subtropical regions that causes vascular wilt in many plant species [60]. Ebihara et al. [61] demonstrated that paddy-upland rotation could completely control the* Verticillium* wilt of eggplant and strawberry. Marenco and Santos [62] showed that the rotation of rice with velvet bean or hyacinth bean reduced weed competition and increased the chlorophyll concentration and yield of rice.* Pseudomonas fluorescens* [63] and* Burkholderia vietnamensis* [64] are well documented as beneficial bacteria in rice fields that increase rice yield and act against sheath blight disease, respectively. As cereal crops are good nematode hosts, whereas legume crops are resistant to this parasite, the yield of rice grain when grown after cowpea and/or mungbean is significantly higher than that after cereal crops [65].

The prevention effect of a specific rotation on pest species proliferation may be mediated by impeded drainage and tillage practices, particularly no-till in which plant residues remain on the field [65]. However, soil microorganisms may also include plant pathogens or deleterious rhizosphere microorganisms, which may negatively affect plant growth and yield [66]. For instance, in the rice-wheat cropping system, some of the diseases and insect pests of rice, such as stem borers, stalk rot, and leaf blight, have occurred in wheat [67, 68]. Thus, the effects of paddy-upland rotation on soil properties have advantages and disadvantages; the utilization of such advantages and the control of the disadvantages will be the focus of future research.

## 5. Conclusions

Paddy-upland rotation is an unavoidable cropping system for Asia to meet the increasing demand for food. However, conventional practices of growing rice and wheat not only deteriorate soil physical properties and decrease water and fertilizer use efficiencies but also cause a stagnation or even reduction in grain yields. Maintaining soil quality at a desirable level is a very complicated and difficult task, because paddy-upland rotation fields are unique from other wetland or upland soils; they are associated with frequent cycling between wetting and drying under anaerobic and aerobic conditions; such rotations change the soil C and N cycles and make the chemical speciation and biological effectiveness of soil nutrient elements varied with seasons, increase the diversity of soil organisms, and make the change of soil physical properties more complicated (Figure 2). Therefore, fully understanding the characteristics of different paddy-upland rotation and its soil properties is necessary in maintaining soil fertility and plant yields. Although new cultivation techniques and nutrient managements, such as minimum tillage systems and combined application of organic and inorganic fertilizers, have been proposed to overcome these problems, extensive investigation and research on potential adverse effects, long-term impacts, and farmer acceptance are still needed. Regardless of the cultivation or management measures used in paddy-upland rotation, the different responses of paddy and upland seasons and their interactions, the long-term effects, the particularity of regions, the differences of ecological conditions, economic and ecological benefits, operability, and the potential of widely use should be considered. By doing so, the paddy-upland rotation can truly achieve sustainable development.

## Figures and Tables

**Table 1:** Changes in soil fertilizer before and after experiment under different land utilizing types.

Reference	Crop rotation	Before experiment					After experiment				
		Soil depth (cm)	SOC (g kg^−1^)	Total N (g kg^−1^)	Total P (g kg^−1^)	Available P (mg kg^−1^)	Soil depth (cm)	SOC (g kg^−1^)	Total N (g kg^−1^)	Total P (g kg^−1^)	Available P (mg kg^−1^)
[14]	Rice—fallow	0–20	13.4	1.74	0.75	7.5	0–10	19.94	2.10	1.07	9.40
							10–20	20.47	2.14	0.65	11.10
	Rice—wheat						0–10	18.60	1.82	0.59	20.70
							10–20	17.60	1.78	0.89	19.50

[19]	Rice—fallow		15.6	2.53	0.62	/	0–10	12.53	2.46	0.53	/
							10–20	12.88	2.36	0.47	
	Rice—rye grass	0–20	14.8	2.50	0.63	/	0–10	12.35	2.67	0.67	
							10–20	10.79	2.48	0.54	
	Rice—Chinese milk vetch		16.9	2.46	0.66	/	0–10	14.97	2.92	0.59	
							10–20	12.41	2.61	0.49	

[20]	Rice—wheat	0–15	/	1.35	/	8.5	0–15	/	1.40	/	11.40
[39]			3.6	/	/	10.0	0–15	4.10	/	/	6.40
[42]			3.2	/	/	11.8	0–15	2.90	/	/	16.10
[43]			5.3	/	/	/	0–15	3.75	/	/	/

[44]	Rice—wheat	0–20	12.3	1.70	/	5.2	0–5	17.0	1.80	/	8.25
							5–12	16.30	1.56		6.83
							12–24	11.0	1.05		1.44

[45]	Fallow	0–15	11.4	1.28	/	/	0–5	32.31	2.89	/	/
							5–15	16.42	1.61		
	Rice—wheat						0–5	16.36	1.74		
							5–15	9.92	1.17		

[46]	Wheat—maize	0–20	5.4	0.41	/	8.3	0–20	6.50	0.66	/	22.10
								6.70	0.71		25.20

[47]	Soybean—maize	0–15	5.4	0.45	/	/	0–15	6.80	1.25	/	/
	Centro—maize							6.43	0.67		
	Cowpea—maize							4.86	0.47		
	Fallow—maize							5.07	0.53		

The data in the table are obtained from field experiments using traditional tillage method and cultivation techniques without any application of organic fertilizers or crop straws. A part of SOC data is derived from the conversion of SOM (SOM = 1.724 ∗ SOC, SOC: soil organic carbon; SOM: soil organic matter).
